# Comparison of inulin clearance with 2-h creatinine clearance in Japanese pediatric patients with renal disease: open-label phase 3 study of inulin

**DOI:** 10.1007/s10157-021-02133-5

**Published:** 2021-09-25

**Authors:** Osamu Uemura, Kenji Ishikura, Koichi Kamei, Riku Hamada, Masaki Yamamoto, Yoshimitsu Gotoh, Naoya Fujita, Tomoyuki Sakai, Takafumi Sano, Masahiko Fushimi, Kazumoto Iijima

**Affiliations:** 1 Department of Pediatrics, Ichinomiya Medical Treatment & Habilitation Center, 1679-2 Tomida-nagaresuji, Ichinomiya-shi, Aichi 494-0018 Japan; 2 Department of Pediatrics, Kitasato University School of Medicine, 1-15-1 Kitazato, Minami-ku, Sagamihara-shi, Kanagawa 252-0374 Japan; 3 Department of Nephrology and Rheumatology, National Center for Child Health and Development, 2-10-1 Okura, Setagaya-ku, Tokyo 157-8535 Japan; 4 Department of Nephrology, Tokyo Metropolitan Children’s Medical Center, 2-8-29 Musashidai, Fuchu-shi, Tokyo 183-8561 Japan; 5 Department of Pediatrics, Seirei Hamamatsu General Hospital, 2-12-12 Sumiyoshi, Naka-ku, Hamamatsu-shi, Shizuoka 430-8558 Japan; 6 Department of Pediatric Nephrology, Japanese Red Cross Nagoya Daini Hospital, 2-9 Myoken-cho, Showa-ku, Nagoya-shi, Aichi 466-8650 Japan; 7 Department of Pediatric Nephrology, Aichi Children’s Health and Medical Center, 7-426 Morioka-cho, Obu-shi, Aichi 474-8710 Japan; 8 Department of Pediatrics, Shiga University of Medical Science, Tsukinowa, Seta, Otsu-shi, Shiga 520-2192 Japan; 9 Development Department, Research Institute, Fuji Yakuhin Co., Ltd, 4-383, Sakuragi-cho, Omiya-ku, Saitama-shi, Saitama 330-9508 Japan; 10 Department of Pediatrics, Kobe University Graduate School of Medicine, 7-5-2 Kusunoki-Cho, Chuo-ku, Kobe 650-0017 Japan

**Keywords:** Inulin clearance, Creatinine clearance, Glomerular Filtration Rate, Chronic kidney disease, Children

## Abstract

**Background:**

There is no approved dosage and administration of inulin for children. Therefore, we measured inulin clearance (Cin) in pediatric patients with renal disease using the pediatric dosage and administration formulated by the Japanese Society for Pediatric Nephrology, and compared Cin with creatinine clearance (Ccr) measured at the same time. We examined to what degree Ccr overestimates Cin, using the clearance ratio (Ccr/Cin), and confirmed the safety of inulin in pediatric patients.

**Methods:**

Pediatric renal disease patients aged 18 years or younger were enrolled. Inulin (1.0 g/dL) was administered intravenously at a priming rate of 8 mL/kg/hr (max 300 mL/hr) for 30 min. Next, patients received inulin at a maintenance rate of 0.7 × eGFR mL/min/1.73 m^2^ × body surface area (max 100 mL/hr) for 120 min. With the time the maintenance rate was initiated as a starting point, blood was collected at 30 and 90 min, while urine was collected twice at 60-min intervals. The primary endpoint was the ratio of Ccr to Cin (Ccr/Cin).

**Results:**

Inulin was administered to 60 pediatric patients with renal disease; 1 patient was discontinued and 59 completed. The primary endpoint, Ccr/Cin, was 1.78 ± 0.52 (mean ± standard deviation). Regarding safety, five adverse events were observed in four patients (6.7%); all were non-serious. No adverse reactions were observed in this study.

**Conclusions:**

The results in this study on the dosage and administration of inulin showed that inulin can safely and accurately determine GFR in pediatric patients with renal disease.

**ClinicalTrials.gov identifier:**

NCT03345316.

## Introduction

Inulin is uniformly distributed in extracellular fluid, filtered by the renal glomerulus, and then excreted in the urine without being secreted or reabsorbed by the renal tubules due to its physical and chemical properties [1]. In addition, because inulin does not bind to plasma proteins and circulates in the body without being metabolized, inulin clearance (Cin) is regarded as the gold standard for glomerular filtration rate (GFR) determination. However, since the method used to measure Cin is complicated and imposes a heavy burden on children, renal function in pediatric patients is usually evaluated using the estimated GFR (eGFR) calculated from creatinine (Cre), cystatin C (Cys-C) or beta 2-microglobulin (BMG), and creatinine clearance (Ccr). Serum Cre (sCre) is widely used as a renal function marker in clinical practice, but it is known that Ccr overestimates the actual GFR because Cre is excreted in the urine by secretion into renal tubules in addition to glomerular filtration [2]. Furthermore, since Cre is produced in skeletal muscle and sCre is proportional to muscle mass, eGFR calculated from sCre levels does not always accurately reflect the actual GFR in pediatric patients with significantly less muscle mass due to severe psychosomatic disorders, neuromuscular disease, and poor nutrition [3]. For such patients, renal function should be evaluated by eGFR calculated from serum Cys-C (sCys-C) or serum BMG (sBMG). However, it has been reported that sCys-C is increased by hyperthyroidism, human immunodeficiency virus (HIV) infection and steroids, while it is decreased by hypothyroidism, and taking cyclosporine [4–6]. In addition, it has been reported that sBMG is increased by inflammatory diseases, infections, malignant tumors, autoimmune diseases, and hyperthyroidism, while it is decreased by hypothyroidism [7, 8]. If eGFR is assumed to be inaccurate due to the above reasons, or each eGFR is significantly different, Cin, which provides an accurate GFR, will be required.

In clinical practice in Japan, inulin is used to evaluate kidney function in pediatric patients with chronic kidney disease (CKD), but in some cases it is used as a physician-determined method not as something having an approved dosage and administration. Therefore, the Japanese Society for Pediatric Nephrology examined the Cin measurement method, dosage and administration for Japanese pediatric patients, and developed a standard method with reference to the method proposed by Cole [9, 10]. In the standard method, drinking water before and during the test, which is difficult for children, was changed to infusion with Ringer’s solution. Furthermore, considering the difficulties associated with blood collection and the unreliability of urination in children, the number of blood and urine collections was reduced compared to the approved administration. In the standard method, the priming dose was calculated based on patient body weight, and the maintenance dose was calculated from individual eGFR and body surface area (BSA).

Members of the Japanese Society for Pediatric Nephrology conducted a clinical study to investigate any discrepancies between Cin measured by the standard method and 2-hour Ccr (2-h Ccr) measured at the same time [11]. They reported that the median 2-h Ccr and Cin in pediatric patients with renal disease was 121.6 and 74.0 mL/min/1.73 m^2^, respectively, and found that 2-h Ccr may overestimate Cin by approximately 1.5-fold, regardless of the GFR value. In addition, on calculating the Ccr/Cin value in pediatric patients with either CKD stage 2 or 3 using the study data, it was found that the lower limit (2.5th percentile value) of the Ccr/Cin value in 95% of patients excluding the 2.5% above and below the Ccr/Cin value was 1.19 for stage 2 and 1.36 for stage 3, respectively (data not shown). This result indicates that Ccr overestimates Cin by at least 1.2-fold in pediatric patients with CKD stage 2 or 3.

Generally, because pediatric nephrologists perform the Cin test using the standard method, in this study, we measured the Cin in pediatric patients (aged < 18 years) with CKD stage 2 or 3 using a similar method. In addition, with reference to the previous study [11], we verified that 2-h Ccr overestimated Cin by at least 1.2-fold, and confirmed the safety of inulin in pediatric patients.

## Materials and methods

### Study design

**Fig. 1:**
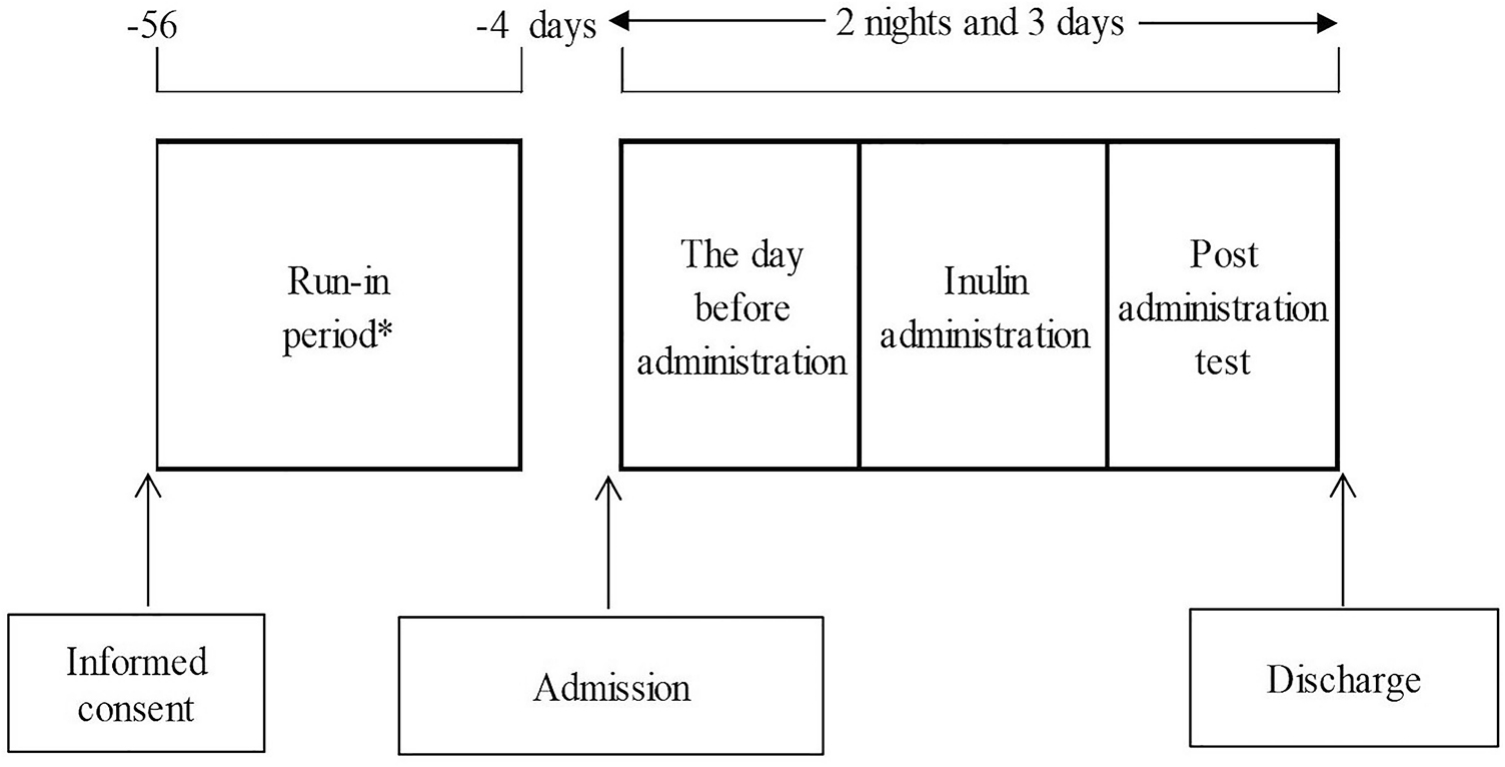
Study schema. *Patients who had been treated with drugs that may affect serum inulin, sCre, sCys-C, and sBMG underwent a wash-out period

This was a phase 3, open-label, single-dose study conducted at six institutions in Japan. Figure 1 shows the study schema. Patients who were judged to be eligible were hospitalized the day before inulin administration and discharged the following day.

### Inclusion and exclusion criteria

Patients who met all inclusion criteria and did not fall under any exclusion criteria were enrolled in the study. Inclusion criteria for this study were as follows: patients with renal disease who require accurate GFR measurement (congenital anomalies of the kidney and urinary tract (CAKUT), reflux nephropathy, nephrotic syndrome, chronic glomerulonephritis, nephronophthisis, neurogenic bladder, polycystic kidney disease, Alport syndrome, etc.); Japanese patients aged 18 years or younger on the day of inulin administration; eGFR 30–89 mL/min/1.73 m^2^ calculated from the following Uemura’s formula using sCre during the run-in period (sCys-C for patients aged under 2 years):

Males: eGFR (sCre) = 110.2 × [(− 1.259 *x*^5^ + 7.815 *x*^4^ − 18.57 *x*^3^ + 21.39 *x*^2^ − 11.71 *x* + 2.628)/sCre] + 2.93 [9]

Females: eGFR (sCre) = 110.2 × [(− 4.536 *x*^5^ + 27.16 *x*^4^ − 63.47 *x*^3^ + 72.43 *x*^2^ − 40.06 *x* + 8.778)/sCre] + 2.93 [9]

Body length (m) is expressed as “*x*”.

eGFR (sCys-C) = (104.1/sCys-C) − 7.80 [12]

Exclusion criteria for this study were as follows: patients who cannot urinate reliably by spontaneous urination, except for catheter urination; patients with edema (excluding those with mild acupressure impression), oliguria, and dehydration the day before inulin administration; patients with diseases that may affect sCre, sCys-C, and sBMG (neuromuscular disease, thyroid dysfunction, autoimmune disease, etc.); patients who used drugs that may affect serum inulin, sCre, sCys-C, and sBMG; patients with infections or inflammatory diseases (excluding chronic diseases) before inulin administration; patients with a history of allergy (including hypersensitivity) to inulin, food, drugs, and metals; patients with any other clinically significant medical conditions that could potentially preclude participation in this study.

### Efficacy endpoints

The primary endpoint was the clearance ratio (Ccr/Cin) between Cin and 2-h Ccr measured at the same time. The following secondary endpoints were set: 2-h Ccr measured at the same time as Cin and eGFR calculated from sCre, sCys-C, and sBMG measured at the same time as Cin. The sCre, sCys-C and sBMG-based eGFR were calculated using the formulas shown below [9, 12–14]:

Males: eGFR (sCre) = 110.2 × [(− 1.259 *x*^5^ + 7.815 *x*^4^ − 18.57 *x*^3^ + 21.39 *x*^2^ − 11.71 *x* + 2.628)/sCre] + 2.93.

Females: eGFR (sCre) = 110.2 × [(− 4.536 *x*^5^ + 27.16 *x*^4^ − 63.47 *x*^3^ + 72.43 *x*^2^ − 40.06 *x* + 8.778)/sCre] + 2.93.

Body length (m) is expressed as “*x*”.

If the patient was younger than 2 years, the above calculation result was multiplied by {0.107 × ln (Age [month]) + 0.656}.

eGFR (sCys-C) = (104.1/sCys-C) − 7.80.

eGFR (sBMG) = (149.0/sBMG) + 9.15.

According to the result of the previous study [11], the 2.5th percentile value of Ccr/Cin in pediatric patients with CKD stage 2 was 1.19. Therefore, the primary endpoint criterion for this study was set to 1.2 to confirm that the 2.5th percentile value of Ccr/Cin for this study was higher than 1.19.

### Safety evaluations

In this study, adverse events (AEs) and safety assessments were conducted by clinical investigators based on vital signs, 12-lead electrocardiography, clinical laboratory tests, and clinical examinations. These were performed during run-in period, before and after inulin administration, and the next day, respectively. AEs were classified according to system organ class and preferred term (MedDRA version 22.1; Japanese Maintenance Organization, Tokyo, Japan) and were evaluated in terms of their potential causal relationship with inulin, as well as severity and seriousness. AEs judged to be related to the study drug were defined as adverse drug reactions (ADRs).

### Dosage and administration

**Fig. 2:**
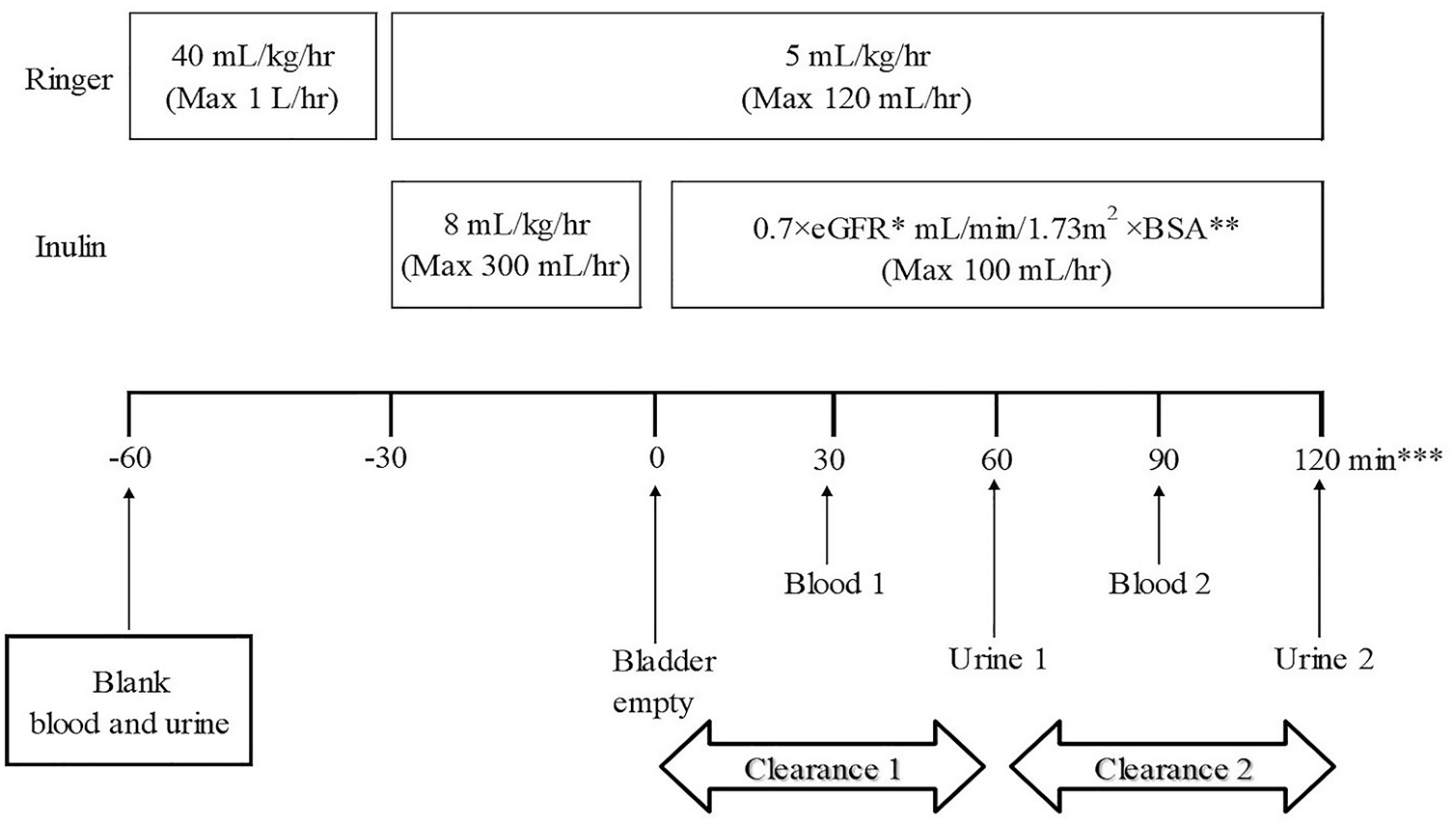
Dosage and administration of inulin. *Calculated using sCre for patients aged 2 years or older and sCys-C for those younger than 2 years. **Calculated using the Haycock method. ***If urination was not observed, the administration duration could be extended to 150 min

Figure 2 shows the dosage, administration, and method used to measure inulin. Patients fasted from at least 4 hours before the start of inulin administration. The inulin concentration was adjusted to 1.0 g/dL in accordance with the package inserted. The priming rate was set at 8 mL/kg/hr (max: 300 mL/hr) for 30 min. Subsequently, the maintenance rate was set at 0.7 × eGFR mL/min/1.73 m^2^ × BSA (max: 100 mL/hr) for 120 min of continuous injection. This eGFR was calculated from the formula using sCre [9] for patients aged 2 years or older and the formula using sCys-C [12] for those younger than 2 years.

If no urine was passed during the first 120 min, the administration period could be extended for up to 150 min.

The dosage and administration of inulin in this study was set in accordance with the standard method formulated by the Japanese Society for Pediatric Nephrology. The target blood inulin concentration at this dosage is set to 20 mg/dL with reference to the method proposed by Cole [10]. The inulin dose required to reach the target blood concentration at the priming dose was calculated to be 40 mg/kg, and the priming rate for 30 min was 8 mL/kg/hr. The maintenance dose was set to an administration rate obtained by the following formula: 0.7 × eGFR mL/min/1.73 m^2^ × BSA taking into account the excretion of inulin in urine. BSA was calculated by the Haycock method [15]. Furthermore, instead of taking water by mouth, which is the usual practice in the approved method, Ringer’s solution was intravenously infused at a rate of 40 mL/kg/hr (max: 1 L/hr) from 30 min before inulin administration began, and thereafter continued at a rate of 5 mL/kg/hr (max: 120 mL/hr) until the end of inulin administration. Thirty minutes after the start of inulin administration, the bladder was emptied by urination, and the time at this point was set to 0. Thereafter, blood samples were collected twice, first at 30 min and then at 90 min. Urine samples were also obtained twice, first at 60 min and then at 120 min.

### Statistical analyses

The efficacy analysis was performed in the full analysis set (FAS), which comprised all patients who met the inclusion criteria and did not fall under the exclusion criteria, who had one or more efficacy endpoints measured, and who had no violations of Good Clinical Practice (GCP).

For the efficacy endpoint, we used the greater of the two calculated values for Cin, calculating the following summary statistics: Ccr/Cin and the two-sided, 95% confidence intervals of the mean value. For the eGFR calculated using sCre, sCys-C, and sBMG and clearance ratio (eGFR/Cin), the two-sided 95% confidence intervals of the summary statistics and mean were similarly calculated.

The safety analysis was performed in the safety population (SP), which comprised all patients who received inulin, for whom safety information is available for evaluation, and who had no violations of GCP.

SAS software, version over 9.2 (SAS Institute, Cary, NC, USA) was used for statistical analysis. The study adopted a two-sided significance level of 5% for all statistical analyses.

## Results

### Patient flowcharts and baseline characteristics

**Fig. 3:**
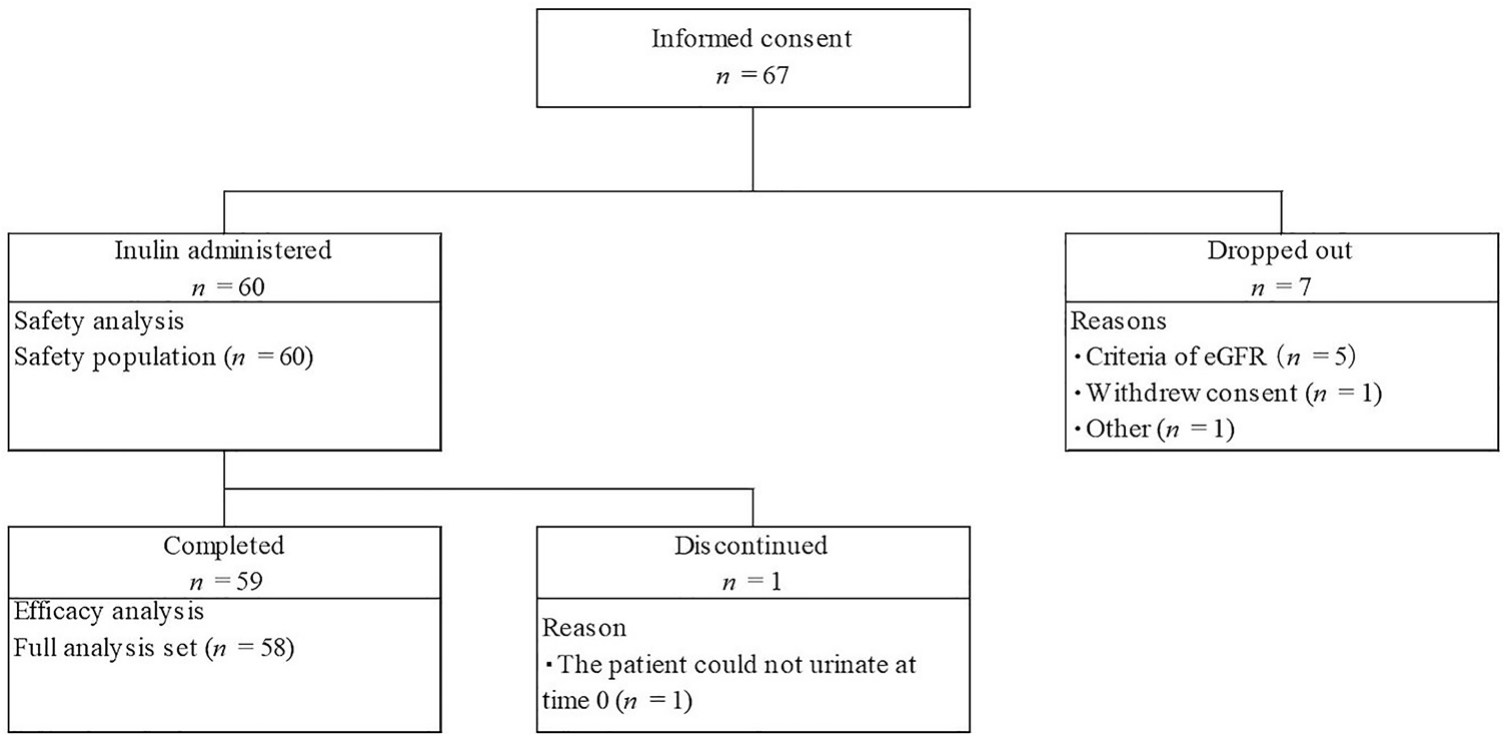
Flow diagram of study protocol

Figure 3 shows the flow diagram of the study protocol. Informed consent was obtained from 67 patients and 7 patients were withdrawn as they no longer met the inclusion criteria or came to fall under the exclusion criteria. Inulin was administered to the 60 patients confirmed to be eligible. Of these 60 patients, 1 patient was discontinued and 59 completed the study. In addition, 58 patients were included in the FAS, excluding 2 patients for whom efficacy data could not be obtained due to discontinuation or incomplete urine collection. The safety population (SP) comprised 60 patients who received inulin. Table 1 shows the baseline characteristics of the patients (FAS) included in this study. There were 36 males (62.1%) and 22 females (37.9%). The height, weight and eGFR (mean ± SD) were 134.71 ± 25.51 cm, 34.36 ± 16.30 kg, and 63.4 ± 15.6 mL/min/1.73 m^2^, respectively. There was 1 patient aged 1 year (1.7%), 30 patients aged from 2 to 11 years (51.7%), and 27 patients aged from 12 to 18 years (46.6%). The most common causative disease was CAKUT in 45 patients (77.6%).

**Table 1:** Baseline characteristics of enrolled patients (FAS)

Characteristic	*n* (%)	Mean ± SD
Sex		
Male	36 (62.1)	
Female	22 (37.9)	
Age (year)		
< 2	1 (1.7)	
≥ 2 and < 12	30 (51.7)	
≥ 12 and ≤ 18	27 (46.6)	
CKD stage		
2	33 (56.9)	
3a	17 (29.3)	
3b	8 (13.8)	
Renal abnormality^a^		
Congenital anomalies of the kidney and urinary tract	45 (77.6)	
Reflux nephropathy	4 (6.9)	
Nephrotic syndrome	1 (1.7)	
Chronic glomerulonephritis	2 (3.4)	
Neurogenic bladder	4 (6.9)	
Alport’s syndrome	2 (3.4)	
Focal glomerulosclerosis	2 (3.4)	
Hemolytic-uremic syndrome	1 (1.7)	
Others	2 (3.4)	
Height (cm)		134.71 ± 25.51
Weight (kg)		34.36 ± 16.30
eGFR (mL/min/1.73 m^2^)		63.4 ± 15.6

^a^If a subject fell into more than one category, they were counted more than once, so the total is greater than the number of cases

### Efficacy

**Fig. 4:**
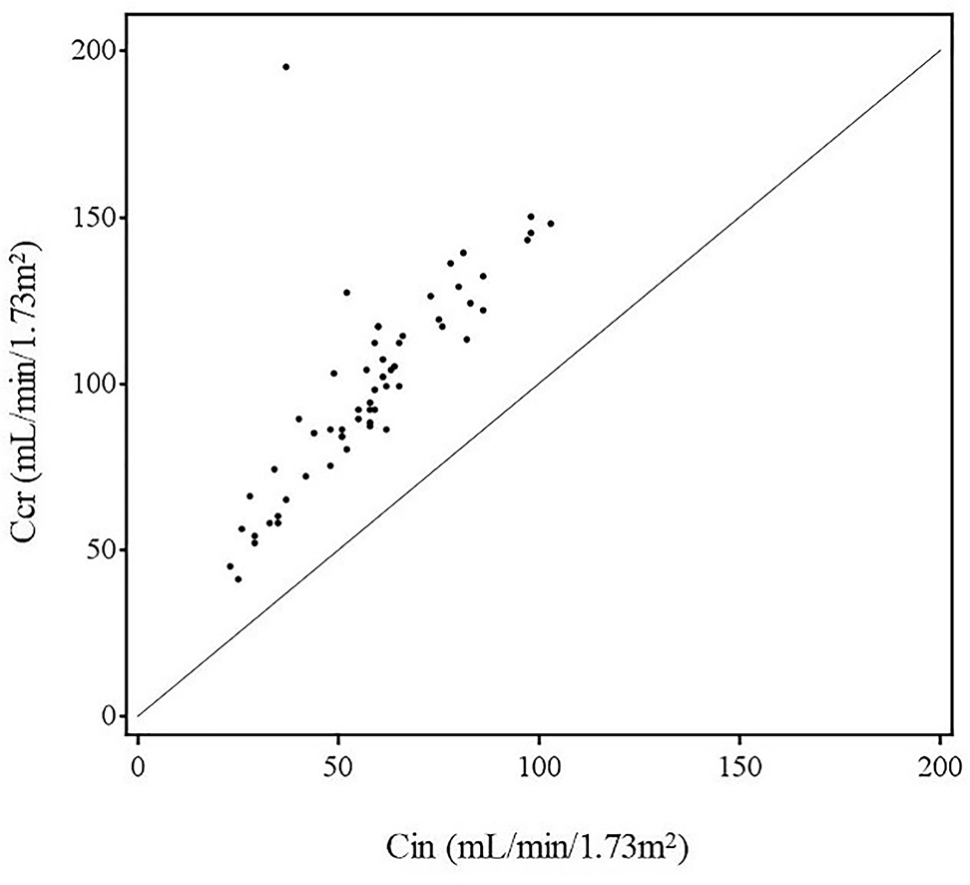
Scatter plot of Ccr and Cin

The Cin and Ccr (mean ± SD), measured at the same time, were 57.9 ± 19.8 and 99.1 ± 30.1 mL/min/1.73 m^2^, respectively. The clearance ratio (Ccr/Cin) between Cin and Ccr was 1.78 ± 0.52 (mean ± SD). In addition, the lower limit of the two-sided 95% confidence interval of the mean value was 1.6406, exceeding the preset criterion of 1.2 (*p* < 0.001, 1-sample *t* test) (Table 2). Figure 4 shows the scatter plot of Ccr and Cin. This result showed that the Ccr exceeded the Cin in all patients.

**Table 2:** Result of Ccr, Cin and Ccr/Cin efficacy endpoints

Parameters	*n*	Mean	SD	95% confidence interval
Ccr	58	99.1	30.1	91.2–107.0
Cin	58	57.9	19.8	52.7–63.1
Ccr/Cin	58	1.78	0.52	1.6406–1.9142
One-sample *t* test^a^	*p* value			*p* < 0.001
	95% confidence interval			1.6406–1.9142

^a^The null hypothesis was “Ccr/Cin = 1.2”

The eGFR (mean ± SD) calculated from sCre, sCys-C, and sBMG measured at the same time as Cin was 68.2 ± 17.7, 79.5 ± 24.3, and 78.5 ± 23.1 mL/min/1.73 m^2^, respectively. The clearance ratios (eGFR/Cin) were 1.24 ± 0.28, 1.42 ± 0.35, and 1.40 ± 0.28 (mean ± SD), respectively (Table 3).

**Table 3:** Result of eGFR and relationship with Cin

Parameters	Parameters used to calculate eGFR	*n*	Mean	SD	95% confidence interval
eGFR	Cre	58	68.2	17.7	63.5–72.9
	Cys-C	58	79.5	24.3	73.2–85.9
	BMG	58	78.5	23.1	72.4–84.6
eGFR/Cin	Cre	58	1.24	0.28	1.1642–1.3103
	Cys-C	58	1.42	0.35	1.3313–1.5170
	BMG	58	1.40	0.28	1.3292–1.4779

### Safety

Table 4 shows all AEs that occurred during the study. The incidence of AEs was 6.7% (four patients), and no serious adverse events were observed. Vomiting and hypoglycemia occurred in one patient were moderate in severity. These two events may have occurred because the patient continued to fast, thus a causal relationship with inulin was ruled out.

**Table 4:** Incidences of adverse events

Adverse events	Safety population (*n* = 60)		
	Number of events	Number of patients	Incidence (%)
All	5	4	6.7
Conjunctivitis	1	1	1.7
Vomiting	1	1	1.7
Injection site pain	1	1	1.7
Hypoglycaemia	1	1	1.7
Presyncope	1	1	1.7

The adverse event name is shown by preferred term per MedDRA version 22.1

Neither severe AEs nor AEs leading to discontinuation of the study were observed. No ADRs were reported.

Accordingly, no major safety issues were observed in any of the pediatric patients with renal disease.

## Discussion

The approved dosage and administration of inulin is set to a uniform dose regardless of body type or renal function. In children, because there is a large difference in body size depending on age, if they were to be administered the same dose as adults, this could lead to an overdose. Therefore, dosage according to the standard method is calculated based on individual body type and level of renal function. In addition, in the approved method, water has to be drunk before and during measurement to ensure that a sufficient amount of urine is generated, but drinking a large amount of water may be difficult for children. Accordingly, infusion of Ringer’s solution is adopted instead of drinking water. The infusion rate of Ringer’s solution is calculated based on body weight and the infusion is continued until the end of inulin administration. Furthermore, considering that the number of blood and urine collections and the intervals between collections in the approved method are also troublesome for children, in the standard method, there are two sample collections at 60-min intervals for both blood and urine.

In this study, Cin in pediatric patients with CKD stage 2 or 3 was measured using a method based on the standard method. The Cin and Ccr (mean ± SD) measured at the same time were 57.9 ± 19.8 and 99.1 ± 30.1 mL/min/1.73 m^2^, respectively. The Ccr/Cin was 1.78 ± 0.52 (mean ± SD), which was confirmed to exceed 1.2. In addition, we compared the results of this study to those obtained in previous study. The previous study showed that the median values of Cin and Ccr were 74.0 and 121.6 mL/min/1.73 m^2^, respectively, and Ccr/Cin was about 1.6. Furthermore, they reported that Ccr/Cin increases as renal function declines [11]. In this study, the median values of Cin and Ccr were 58.0 and 98.5 mL/min/1.73 m^2^, respectively, and the median Ccr/Cin was 1.67. The median Ccr/Cin was calculated to be 1.65, 1.67, and 1.78 for CKD stage 2, 3a, and 3b, respectively, suggesting that Ccr/Cin tended to increase as renal function declined. It has been reported that Ccr/Cin generally tends to increase as renal function declines [16]. Since the results of this study and previous study confirmed this tendency, Cin using the standard method was considered to accurately reflect kidney function.

For patients whose sCre level may not be an accurate reflection of renal function, eGFR using sCys-C or sBMG may be useful [12, 13]. In this study, eGFR (mean ± SD) calculated from sCre, sCys-C, and sBMG measured at the same time as Cin was 68.2 ± 17.7, 79.5 ± 24.3, and 78.5 ± 23.1 mL/min/1.73 m^2^, respectively, and eGFR/Cin (mean ± SD) was 1.24 ± 0.28, 1.42 ± 0.35, and 1.40 ± 0.28, respectively. It was revealed that the eGFR calculated from sCre was the closest to Cin, and the eGFR values calculated from sCys-C and sBMG were similar. These results suggest that although all eGFR values are closer to Cin than Ccr, each eGFR may overestimate renal function compared to Cin. Therefore, we believe that Cin measurement using this standard method is necessary to ensure more accurate evaluation of renal function in pediatric patients with renal disease.

Cin may show a slightly lower value than the original kidney function if the urine volume is not measured accurately due to insufficient urine storage. In this study, we used the greater of the two calculated values for Cin, as does the standard method due to concerns about insufficient urine collection. However, it is unclear whether sufficient urine could be collected from pediatric patients, particularly from infants. Therefore, the possibility cannot be ruled out that eGFR/Cin tended to be slightly higher than in the original study and some differences may be observed because eGFR is not affected by urine volume. Previous study observed slight differences between eGFR and Cin [17]. Additionally, although the pediatric GFR estimation formula was created by measuring Cin in patients with CKD stage 1 to 5, many patients with mild renal dysfunction were included. On the other hand, this study only included patients with CKD stage 2 and 3. This difference in patient background may have caused discrepancy in Cin and eGFR. To verify how much the discrepancy between Cin and the eGFR from formula, a comparative study with many patients is required.

Regarding the safety of this study, none of the patients developed edema due to the infusion of Ringer’s solution. No ADRs, serious adverse events, or discontinuation due to adverse events were observed. In short, there were no safety issues.

In summary, the method used to measure Cin in this study provides clinicians with a simpler measurement method for pediatric renal disease patients than the approved method, making it easier to evaluate renal function accurately through the use of Cin. For pediatric patients with renal disease, if evaluation of renal function by Ccr or eGFR is not an option, evaluation of renal function using the Cin measurement method proposed in this study will make it possible to determine treatment strategies such as appropriate drug selection and management of complications.

## Limitations

This study was conducted on a small number of cases with just one patient under 2 years of age. In addition, patients with CKD stage 2 and 3 were targeted, and most of the renal abnormalities were CAKUT.
